# Neuropsychological Outcome after Complicated Shiga Toxin–Producing *Escherichia coli* Infection

**DOI:** 10.1371/journal.pone.0103029

**Published:** 2014-07-22

**Authors:** Olga Simova, Gabriele Weineck, Thorsten Schuetze, Karl Wegscheider, Ulf Panzer, Rolf A. K. Stahl, Christian Gerloff, Tim Magnus

**Affiliations:** 1 Department of Neurology, University Medical Centre Hamburg-Eppendorf, Hamburg, Germany; 2 Institute of Biometrics, University Medical Centre Hamburg-Eppendorf, Hamburg, Germany; 3 Department of Internal Medicine III and Clinics, University Medical Centre Hamburg-Eppendorf, Hamburg, Germany; University of Münster, Germany

## Abstract

**Background:**

The diarrhea associated hemolytic uremic syndrome (HUS) is a major cause of acute uremic failure in children, but not very common in adults. The enterohaemorrhagic *Escherichia coli* -epidemic in Germany in 2011 affected mostly young and healthy adults. While their immediate deficits have been published, not much is known about the time course and degree of recovery concerning cognitive and behavioral impairment.

**Methods and Findings:**

Twenty patients with Shiga toxin –producing *Escherichia coli* infection and neurological symptoms underwent comprehensive neuropsychological assessment 3 months and 1 year after the acute disease. Overall, there was an excellent recovery of cognitive functions. In a detailed neuropsychological analysis no significant deficits could be noticed 1 year after the infection in terms of cognitive function, alertness, executive functions and speech. Interestingly there were no correlations between different indicators for severity of disease (hemoglobin and creatinin levels, days of hospitalization, neurological symptoms and MRI changes) and neuropsychological outcome. However, there were a small number of patients with limitations in every day and professional life even one year after the acute disease.

**Conclusions:**

Our study does not provide definitive answers regarding risk factors for these limitations. Still since Shiga toxin –producing *Escherichia coli* infection is a rare condition in adults, the information this study provides is important for the clinical practice. On one hand for consulting patients and on the other to raise the awareness of the physicians to possible long term complains and the consideration of neuropsychological assessment and supportive psychological treatment.

## Introduction

The diarrhea associated hemolytic uremic syndrome (HUS) is a major cause of acute uremic failure in children, but not very common in adults [1], [2]. The northern German outbreak of the hypervirulent enteroaggregative Shiga toxin producing *Esherichia coli* (STEC) strain O104 in 2011 involved 3861 patients, 846(22%) of which developed HUS. 88% of the HUS affected patients were adults [3]. Neurological complications were often and severe. In three hospitals in Hamburg 217 patients were treated with complicated STEC infection, of whom 104 (48%) developed neurological symptoms. They ranged from delirium and coma to epileptic seizures, aphasia, apraxia, and other focal deficits as hemiparesis and occulomotor dysfunction. Fortunately most of the patients improved over time and showed no significant neurological deficits at the time of discharge from the hospital [4].

There are investigations in children after HUS with neurological symptoms, which show trends for impairment in behavior, verbal intelligence, and verbally based skills of reading comprehension and vocabulary [5]. Children with HUS and without apparent neurological symptoms do not show any difficulties with learning, behavior or attention when compared to matched controls [6].

The Enterohaemorrhagic *Escherichia coli*-epidemic in Germany affected mostly young and healthy adults. They were abruptly torn from normal life. While their immediate deficits have been published, not much is known about the time course and degree of recovery concerning cognitive and behavioral impairment [4]. Therefore, we followed patients with STEC infection and neurological symptoms and assessed cognitive and behavioral impairment after the acute phase of disease.

## Methods

### Study design and participants

The study was carried out at the university medical center Hamburg Eppendorf during the STEC outbreak in spring 2011. All patients with (1) confirmed STEC infection, with or without HUS, who developed (2) neurological symptoms verified by a senior neurologist were included. Out of 60 patients who met these criteria, 20 were able and agreed to undergo a comprehensive neuropsychological evaluation. The data collection followed the guidelines of the Hamburg Board of Physicians and was approved by the Ethics Committee of the Hamburg Board of Physicians. All participants provided their written informed consent for participation in the study and the Ethics Committee of the Hamburg Board of Physicians approved the consent procedure.

### Cognitive and behavioral assessments

Neuropsychological assessment included comprehensive test battery for evaluation of (1) memory, (2) attention and speed and (3) executive function and verbal ability. Table 1 gives an overview of the neuropsychological domains and tests. All tests depicted in table 1 are based on objective rating system [7].

**Table 1:** Neuropsychological domains and tests.

Domain/Test	Subtest	Description	Reference
**Memory**			
Wechsler memory scale (WMS-R)	Working memory numbers	Digit span of working memory	[11]
Verbal learning memory test (VLMT)	VLMT, VLMT-DG5, VLMT-DG 1-5, VMLT-proactive and retroactive interference, VLMT-DG5-DG7, VLMT-A W-F	Immediate memory, word span learning, New verbal learning course, Susceptibility of interference, Retention of verbal information and memory recognition	[12]
Rivermead behavioral memory test (RBMT)	Story recall subtest	Short and long term logical verbal memory	[13]
**Attention/Speed**			
TAP 2.1	Alertness, Selective Attention (GoNoGo1), Split attention	Reaction time and accuracy	[14]
Trail making Test (TMT)	TMT A	Visuo-motor speed and visual selection	[15]
Color-Word Interference Test (FWIT)	Color naming	Cognitive processing speed	[16]
**Executive functions/Speech**			
Phonematic and semantic verbal fluency test (RWT)	RWT K, RWT Animals	Verbal fluency	[17]
Trail making Test (TMT)	TMT B	Mental flexibility	[15]
FWIT-selective attention	Difference List 3-List 2	Focused attention	[16]

Mood and anxiety were evaluated by the The Hospitality Anxiety and Depression Scale (HADS-D) and cognitive and physical fatigue by the Wuerzburg Fatigue Inventory in multiple sclerosis (WEIMuS) [8], [9]. Psychological complains were recorded using the symptom checklist SCL-90-R [10]. HADS-D, WEIMuS and SCL-90-R are questionnaires and reflect the subjective estimation of the patients.

### Procedures

All patients were examined by a senior neurologist to confirm neurologic deficits. The neuropsychological testing was carried out by experienced neuropsychologist 10 to 30 weeks after disease onset in a single visit and required approximately 1.5–2 hours. Twelve months later only selected tests with poor performance in the first assessment were repeated in a single visit and required 15–30 minutes, when available parallel test versions were used. At both time points patients were asked to evaluate their recovery in percent compared to healthy condition and to specify the ongoing deficits.

Relevant medical background information, considering neurological deficits, treatment (dialysis, medication), hospitalization and laboratory data were obtained in order to determine the severity of disease.

### Data analysis

Unpaired t-tests and Fischer's exact test were used to compare data between participants and nonparticipants. Paired t-tests were carried out on laboratory data at different time points for the participants group. *P*-value≤0.05 was considered as statistically significant. The neuropsychological scores were given in percentile ranks (PR). This allows a comparison to the normal population with similar background (age, education level). PR values ≥98 indicate very superior, PR 85–97-superior, PR 71–84 high average, PR 30–70 average, PR 16–29 low average, PR 3–15 under average and PR values ≤3 far below average performance of the test taker when compared to the normal population. Kendall's Tau and Spairman's rank correlations were used to analyze the relationship between disease severity parameters and cognitive assessment scores.

## Results

### Group characteristics

73.3% (44) of our 60 patients with neurological symptoms were seen by neurologist after discharge from the hospital, 8.3% (5) died; two due to septic complications, one due to heart failure, one due to complications of status epilepticus and one due to complications of new diagnosed ovarian carcinoma, 1.6% (1) of them had severe neurological deficits (impaired consciousness, cortical blindness, aphasia, apraxia) and non-reversible MRI changes; and were moved to rehabilitation hospital and 16.7% (10) did not attend the regular neurological ambulant check-ups. All 44 patients who attended the regular neurological check-ups were offered a detailed neuropsychological testing. 45% (20) of them underwent neuropsychological testing 10 to 30 weeks after disease onset. 9% (4) refused to participate although admitting cognitive impairment and 40.9% (18) considered themselves as completely recovered and refused participation.

Table 2 provides information about age, gender, neurological deficits and treatment in the participants and nonparticipants-group. The study group was significantly younger in comparison to the nonparticipants (*p*<0.001). There was no significant difference between the groups in terms of sex, occurance of different neurological symptoms and treatment (dialysis, antiepileptic drugs, eculizumab). MRI was performed at equal rates in both groups and the occurrence of pathological findings was significantly higher in the participant-group, but when compared in terms of specific changes, there was no difference between the groups. Table 3 summarizes the severity of disease in both groups using laboratory parameters. There was no significant difference between the groups regarding hemoglobin, leucocytes, thrombocytes, urea, creatnine clearance, CRP and LDH levels. The study group had significantly higher creatinine levels (*p* = 0.02) during acute disease indicating more severe acute renal injury. All laboratory parameters had improved significantly at the time of the first neuropsychological evaluation in comparison to the worst levels within the study group. However, 60% of the patients had lower hemoglobin (9–12 g/dl) and 25% had increased creatinin levels (>3 mg/dl) at this time point. One year later all patients had reached normal hemoglobin levels but the mentioned 25% still had increased creatinin levels and low creatinine clearance. There were no significant differences between participants and nonparticipants concerning laboratory values although there was a tendency for lower creatinine clearance in the participant-group.

**Table 2:** Characteristics of the study patients.

	Participants (n = 20)	Non-participants (n = 40)	*p*-Value
Age mean/±SD years	38±14.5	51.5±19.7	0.001*
Sex male/female %	25/75	30/70	0.466
Headaches %	25	12.5	0.135
Acute confusional state %	75	75	0.617
Aphasia %	35	40	0.466
Paresis %	15	15	0.296
Oculomotor dysfunction %	5	12.5	0.263
Apraxia %	20	30	0.181
Epileptic seizures %	45	30	0.194
Myoclonus %	25	30	0.224
Intubation %	40	22.5	0.133
Dyalisis %	85	85	0.296
Levetiarcetam %	85	80	0.464
Eculizumab %	75	65	0.466
Cerebral MRI %	80	70	0.308
MRI pathological findings	75	43	0.039*

*- statistically significant.

**Table 3:** Laboratory data.

	Worse during disease			1. neuropsych. test		1 year after acute disease		
	Part.	Nonpart.	*p*	Part.	*p* (vs worse)	Part.	Nonpart.	*p*
Lowest Hemoglobin (g/dl)	7±1.2	7±1.3	0.27	12±1.4	5.69 E-12*	13.6±1.1	13.3±1.3	0.41
Highest Leucocytes (x10^9^/l)	22±13.1	20±9.8	0.42	5±2	2.69 E-05*	6.4±1.4	5.9±1.9	0.49
Lowest Thrombocytes (x10^9^/l)	53±30.5	43±34.8	0.31	246±67.7	2.72 E-09*	249.4±54.9	252.9±79.5	0.87
Peak urea (mg/dl)	73±32	70±29.4	0.71	22±21.3	7.85 E-06*	16.4±4.0	20.0±9.0	0.44
Peak creatinine (mg/dl)	7±3.8	4±2.6	0.02*	1.2±0.5	4.32 E-06*	0.9±0.2	1.1±0.5	0.29
Lowest creatinine clearance (ml/min)	16±16.1	22±17.6	0.21	53±11.4	3.58 E-09*	59.2±3.7	54.3±10.9	0.06
Highest CRP (mg/l)	149±113.9	156±193.7	0.78	2±2.3	5.37 E-05*	5±0	5±0	1
Highest LDH (U/l)	1367±654.3	1318±820	0.82	205±30.1	2.55 E-07*	202.7±41.0	185.3±24.6	0.11

CRP – C-reactive protein;

LDH – Lactate dehydrogenase,

*p* – p Value,

*- statistically significant.

At the time point of the first neuropsychological evaluation 40% of the study patients still received eculizumab and 25%-antiepileptic drugs (15% levetiracetam and 10% phenytoin), nobody was on dialysis, and MRI-changes were not detectible anymore. One year later none of the patients was treated with eculizumab or antiepileptic drugs. 95% of the patients were normal on standard neurological examination. One patient had visual field defects in the lower nasal fields on both sides after retinal hemorrhages at both time points of assessment.

### Cognitive and behavioral outcomes

At the time point of the first neuropsychological evaluation all participants had been discharged from our hospital. 8/20 had returned to their normal everyday-or professional life. 3/20 worked part-time and 9/20 were certified unfit for work. The subjective recovery ranged from 55 to 95% (78±12, 5). 18/20 reported fatigue/slowing, 13/20 - somatic complains (i.e. gastro-intestinal complains, knee/shoulder pain, headaches, visual field deficit), 12/20- lack of concentration and 4/20 speech difficulties.

One year later the subjective recovery ranged from 70 to 100% (90±8.8) and was significantly better in comparison to the first evaluation (p<0.005). Two patients did not attend the second neuropsychological assessment (18/20). One attended the neurological check up and answered questions about subjective recovery (19/20), the other did not. Both of them were working/studying full time. 17/20 patients worked full time or had returned to normal life without limitations, 3/20 worked part time because of ongoing fatigue and difficulties to concentrate. Nevertheless, 5/19 patients reported fatigue/slowing, 8/19 concentration problems, 8/19 somatic complains (kidney problems, one patient had continuing visual field deficit after retinal hemorrhage). Only the patients who presented for both tests were included in the statistical follow up analysis (N18). All 20 participants were included in correlation studies.

The results of the comprehensive neuropsychological evaluation are summarized in Table 4 and 5. In the first neuropsychological evaluation 1/18 patient performed better in all subtest than under average (>15 PR), 9/18 participants achieved borderline or lower scores (≤15 PR) in two or less different subtests, 7/18 –in three or four subtests and only one patient- in seven subtests. In accordance with the subjective complains the highest rate of scores below 15 percentile ranks was in the alertness and velocity domain. In the second assessment only selected tasks with poor performance (≤15 PR) in the first evaluation were repeated. 11/18 patients had better than under average (>15 PR) performance in all subtests, 6/18 achieved poor scores (≤15 PR) in two or less subtests, one patient in three subtests and another patient- in five subtests. Looking at individual level the test takers performed better in 27 out of 37 subtests in the second evaluation (13 significantly better, 14 numerical), in 10/37 subtests they performed equal and only one patient performed worse in three subtests for attention and speed in the follow up.

**Table 4:** Number of subtests with PR<15.

	Number of subtests with PR<15																			
Patients	P1	P2	P3	P4	P5	P6	P7	P8	P9	P10	P11	P12	P13	P14	P15	P16	P17	P18	P19	P20
**1. evaluation**																				
Memory	0	0	1	0	0	2	3	0	0	2	0	**0**	0	0	0	0	**3**	0	0	0
Attention/Speed	0	2	2	1	1	2	2	1	2	1	3	**1**	0	0	0	1	**0**	1	0	2
Executive functions/Speech	3	1	1	0	0	0	2	0	0	0	1	**0**	1	1	0	0	**1**	0	1	1
**Total**	3	3	4	1	1	4	7	1	2	3	4	**1**	1	1	0	1	**4**	1	1	3
**2. evaluation**																				
Memory	*0*	*0*	0	*0*	*0*	0	0	*0*	*0*	1	*0*	**n.c**	*0*	*0*	*0*	*0*	**n.c**	*0*	*0*	*0*
Attention/Speed	*0*	0	3	0	0	1	2	0	0	1	0	**n.c**	*0*	*0*	*0*	0	**n.c**	0	*0*	1
Executive functions/Speech	1	0	0	*0*	*0*	*0*	2	*0*	*0*	*0*	1	**n.c**	0	0	*0*	*0*	**n.c**	*0*	0	0
**Total**	1	0	3	0	0	1	5	0	0	2	1	**n.c**	0	0	0	0	**n.c**	0	0	1

n.c. =  not controlled.

Table 4 depicts the number of subtests with poor performance (≤15 PR) for each neuropsychological domain and every single patient for both time points: 1. evaluation - 3months and 2. evaluation - 1 year after acute disease. In the second evaluation only selected tests with under average performance (≤15 PR) in the first assessment were repeated. All not-repeated subtests with scores >15 PR in the first testing are considered as *0* in the second one.

**Table 5:** Total number of subtests with PR<15 (18 participants).

	1. neuropsych. evaluation	2. neuropsych evaluation
Memory	8	1
Attention/Speed	21	8
Executive functions/Speech	12	4

Table 5 shows the total number of subtests with poor performance (PR<15) for each neuropsychological domain for both time points- 3months and 1 year after acute disease. Only patients who accomplished the assessment at both time points were included (18 of 20 patients, as shown in table 4).

In the first evaluation only one patient showed abnormalities in terms of anxiety symptoms assessed by the HADS-D self-report scale. Three patients had higher levels of psychological problems when compared to the normal population (SCL-90 R PR>84) and three declared high intensity of fatigue symptoms using the *WEIMuS* self-report scale (>37 P). One patient acknowledged anxiety, psychological problems and fatigue. One year later the same patient reported continuing complains (HADS-A, *WEIMuS*) and another patient, who previously did not have psychological problems showed increased scores for fatigue (*WEIMuS*). Interestingly, the patient with ongoing anxiety and fatigue was the only one who performed worse in the second evaluation of attention and speed.

### Correlations

Although the first neuropsychological assessment was performed at different time points after disease onset (10–30 weeks) there was no correlation between the time point and psychometric measures. Further, we looked for correlations between different indicators for severity of disease (hemoglobin and creatinin levels, days of hospitalization, neurological symptoms and MRI changes) and neuropsychological outcome. There was no association between the highest recorded creatinine concentration or creatinine values at time of testing and poor neuropsychological scores. Similarly, hospitalization (in days) did not affect the cognitive outcome. There was even a tendency for better cognitive performance after longer hospitalization (Spearman-Rho 0.48 for VMLT-A proactive and retroactive interference subtest). Patients with MRI-changes had lower scores in one subtest for selective attention (Alertness TAP 2.1 with auditory stimulus). The occurrence of epileptic seizures during acute disease was associated with lower scores in the RBMT-story recall subtes (Spearman-Rho −0.65) but only one patient performed under average level. In contrary, treatment with levetiracetam and phenytoin correlated with worse performance in single subtests (VLMT-DG5, RBMT-story recall subtest and RWT Animals). Eculizumab medication was associated with better performance in one subtest for executive function (FWIT difference List3–List2).

## Discussion

Central nervous system involvement is common in children and adults with STEC-HUS. Multiple mechanisms are considered to be involved in central nervous system damage including hypertension, hyponatremia, microangiopathy and direct toxic effects of uremia and verotoxin/Shiga-like toxin. However, severe chronic neurologic impairment is reported only in small number of STEC-HUS survivors. During the STEC outbreak in northern Germany in 2011 48% of the STEC infected patients developed neurologic symptoms and only 3% had long term neurological sequelae [4]. While acute neurologic involvement is well described, not much is known about milder cognitive and behavior impairment beyond the acute STEC infection in adults. Schlieper et al described a trend for post-HUS deficit in verbal intelligence and the verbally based skills of reading comprehension and vocabulary, as well as in behavior in children with neurological symptoms during acute disease [5]. To our knowledge there is no information about similar neuropsycholgical effects after acute STEC-HUS disease in adults. The question is very relevant since the STEC epidemic in Germany affected mostly young adults leading to very severe disease which could have severe neuropsychological long term effects and could interfere with their capability to work.

Since a neuropsychological evaluation is a time consuming (1, 5 to 2 hours) procedure which requires concentration, it was not surprising that many potential study-patients refused to participate after weeks-long hospitalization and severe disease. Certainly the small number of participants and the lack of control group and baseline levels of the applied neuropsychological tests were the main limitations of the study. However, the participant-group represents patients who were assumed to keep long-term sequelae after the STEC infection. This assumption was supported by the fact that the participants had higher levels of peak-creatinine, higher incidence of MRI-changes in comparison to the non-participants.

The neuropsychological evaluation confirmed the subjective complains of the participants. Most of the patients reported fatigue/slowing and lack of concentration and looking at all subtests with borderline or lower performance, patients performed particularly badly in areas of attention and speed. In accordance with the subjective estimation, the performance in the second evaluation was significantly better. Only one patient performed worse and below average in one test for alertness and velocity and the same patient reported continuing complains in terms of anxiety and fatigue (HADS-A, WEIMuS).

Our results suggest that the cognitive and behavioral outcome does not depend on the severity of disease measured in peak creatinine levels, hospitalization, and neurological complications. These findings are consistent with previous studies in children [6]. Furthermore, the outcome is not linked to characteristic MRI-changes. So there is no evidence that STEC-HUS infection with reversible neurological symptoms leads to specific cognitive or behavioral deficits. We looked at other possible influences, like treatment with antiepileptic drugs and eculizumab. There was a tendency that treatment with antiepileptic drugs and the occurrence of epileptic seizures are associated with lower scores in some subtests for short and long term memory. However, our study is limited by the lack of individual baseline- levels and the fact, that only a few patients performed below average and that the correlations are restricted to single subtests makes it difficult to determine true correlation.

Irrespective of the performance in the neuropsychological assessment, one should point out that 3/20 participants worked part-time one year after the acute disease, 5/19 still reported fatigue and 8/19 - concentration difficulties. So in spite of good neurological and cognitive recovery some patients seem to have long term difficulties in everyday activities.

Overall, the results are encouraging since even severely affected patients with reversible neurological complications during the acute STEC infection have in general good outcome not only in terms of neurologic symptoms as reported by Magnus et al. but also in terms of cognitive and behavioral functions [4]. Nevertheless, there were a small number of patients with long term limitations in every day and professional life. Our study does not provide definitive answers regarding risk factors for these limitations. However, STEC-infection is a rare condition in adults and the information this study provides is important for the clinical practice. On one hand for consulting patients and on the other to raise the awareness of the physicians for possible long term complains and the consideration of neuropsychological assessment and supportive psychological treatment.
